# Randomised control trial of a proactive intervention supporting recovery in relation to stress and irregular work hours: effects on sleep, burn-out, fatigue and somatic symptoms

**DOI:** 10.1136/oemed-2021-107789

**Published:** 2022-01-24

**Authors:** Anna Dahlgren, Philip Tucker, Majken Epstein, Petter Gustavsson, Marie Söderström

**Affiliations:** 1 Department of Clinical Neuroscience, Karolinska Institute, Stockholm, Sweden; 2 Department of Psychology, Swansea University, Swansea, UK; 3 Department of Psychology, Stockholm University Stress Research Institute, Stockholm, Sweden; 4 Liljeholmens akademiska vårdcentral/Academic Primary Healthcare Center Liljeholmen, Stockholm, Sweden; 5 Department of Psychology, Karolinska Institutet Department of Clinical Neuroscience, Stockholm, Sweden; 6 Stressmottagningen, Stockholm, Sweden

**Keywords:** fatigue, occupational health, sleep, preventive medicine, burnout, psychological

## Abstract

**Objectives:**

To examine if a proactive recovery intervention for newly graduated registered nurses (RNs) could prevent the development of sleep problems, burn-out, fatigue or somatic symptoms.

**Methods:**

The study was a randomised control trial with parallel design. Newly graduated RNs with less than 12 months’ work experience were eligible to participate. 461 RNs from 8 hospitals in Sweden were invited, of which 207 signed up. These were randomised to either intervention or control groups. After adjustments, 99 RNs were included in the intervention group (mean age 27.5 years, 84.7% women) and 108 in the control group (mean age 27.0 years, 90.7% women). 82 RNs in the intervention group attended a group-administered recovery programme, involving three group sessions with 2 weeks between each session, focusing on proactive strategies for sleep and recovery in relation to work stress and shift work. Effects on sleep, burn-out, fatigue and somatic symptoms were measured by questionnaires at baseline, postintervention and at 6 months follow-up.

**Results:**

Preventive effect was seen on somatic symptoms for the intervention group. Also, the intervention group showed less burn-out and fatigue symptoms at postintervention. However, these latter effects did not persist at follow-up. Participants used many of the strategies from the programme.

**Conclusions:**

A proactive, group-administered recovery programme could be helpful in strengthening recovery and preventing negative health consequences for newly graduated RNs.

**Trial registration number:**

NCT04246736.

**Key message**

**What is already known about this subject?**

- Entering work life as a newly graduated registered nurse is stressful.
- Newly graduated registered nurses have a high prevalence of burn-out.
- Recovery has been suggested as a key factor for preventing ill health due to stress.

**What are the new findings?**

- A proactive recovery intervention was shown to be feasible in a working life context, promoting beneficial strategies for sleep and recovery.
- Supporting recovery was associated with positive results on health and well-being.

**How might this impact on policy or clinical practice in the foreseeable future?**

- Further development of methods for supporting employees sleep and recovery is important.
- A proactive approach might be important for managing employee health.

## Introduction

Work, and especially demanding work situations, leads to effort expenditure and a need for recovery^1^ that is signified by the manifestation of fatigue.^2^ Recovery is the process of psychophysiological unwinding after effort, in which mental and physiological resources are replenished.^3^ According to the effort-recovery theory,^1^ recovery is crucial for preventing adverse health consequences due to stress exposure.^4^

There are multiple paths linking insufficient recovery with ill health. Sleep is essential for physiological and psychological recovery, and chronic sleep deprivation can contribute to the development of both somatic and psychological symptoms and ill health, for example, burn-out, depression, cardiovascular disease, etc.^5 6^ While stress is a potential cause of disturbed sleep,^7^ sleep deprivation can itself be a stressor contributing to allostatic load.^8^ According to the allostatic load theory, repeated or prolonged stress exposure can have negative effects on health. Stress reactions can also be sustained after the actual stressor has subsided, through perseverative cognition in the form of worries or rumination.^9^ Difficulties letting go of stressful thoughts, together with high work demands and insufficient sleep, have been shown to predict clinical burn-out.^5^ Hence, perseverative cognition could be one mechanism which, if sustained, may lead to health problems.

Paradoxically, while situations with high work demands featuring high stress levels increase the need for recovery, those are also situations in which recovery is likely to be impaired, a phenomenon referred to as the ‘recovery paradox’.^10^ Impaired recovery during stressful periods could be due to either sleep impairments, failure to detach from thoughts of work during free time, or lack of recovery behaviours such as physical or social activities during leisure time. Work-induced fatigue during free time, which is common during stressful work periods, may further limit the possibilities to engage in beneficial recovery behaviours, and thus contribute to the recovery paradox.

Sleep is not only affected by stress but is also regulated by homoeostatic and circadian factors. The homoeostatic regulation of sleep means that the neurophysiological drive for sleep increases with time awake.^11^ Circadian rhythms make sleep difficult during daytime, when melatonin is low and metabolism is high. For shift workers this often means that they have to initiate sleep at times that are biologically suboptimal. Disturbed sleep is common among shift workers, and is one of the possible mechanisms behind the increased risk of of both somatic and psychological health problems among shift workers.^6^

In order to optimise employees’ health and work performance, organisations should seek to minimise work stressors and promote work hours that enable sufficient sleep and recovery. On an individual level, organisations can encourage employees to adopt beneficial strategies for recovery. Sleep and sleep-related outcomes can be improved by such interventions, with the most common being educational interventions that focus on sleep hygiene and fatigue management.^12^ Cognitive–behavioural therapy for insomnia (CBT-I) has been shown to be effective among adults in the general population.^13^ However, shift workers face more demanding challenges in managing sleep in relation to irregular work hours. Group-administered CBT-I for shift workers, including sleep hygiene, relaxation, cognitive restructuring, etc, have shown improvements in sleep outcomes, although a follow-up study did not show that CBT-I was better than a sleep hygiene programme.^14 15^

Few studies have examined interventions aimed at promoting recovery in forms other than sleep. Supporting recovery behaviours in workers with high levels of stress symptoms was found to reduce stress and burn-out, as well as depressive and anxiety symptoms.^16 17^ Recovery behaviours were defined as appetitive behaviours supporting psychophysiological detachment following exposure to stressors or effort expenditure. Participants were encouraged to try various such behaviours in different contexts for example, listening to music, engaging in physical activity, etc.

Entering working life is a period often characterised by high stress for registered nurses (RNs), described as a reality, or transition, chock.^18^ Besides the high workload and the stress of being new in the professional role, many RNs also start working shifts, which is a risk factor for impaired sleep. New RNs may often lack effective strategies for managing sleep and fatigue, and the strategies used may sometimes be counterproductive.^19 20^ RNs also have a high prevalence of burn-out and somatic symptoms early in their career.^21 22^

Given the challenges facing new RNs, actions are needed to protect the processes of recovery and thereby buffer the impact of their stressful work situation. The objective of the current study was to examine whether a proactive intervention, a group-administered recovery programme focusing on promoting strategies for sleep and recovery, could mitigate the impact of work stress and shift work and thus prevent the development of sleep problems, burn-out, fatigue and somatic symptoms among new RNs. The intervention focused on three main themes: (1) unwinding from stress; (2) promoting sleep according to homoeostatic and circadian factors; and (3) handling fatigue by increasing recovery behaviours.

There were seven primary outcomes, namely: two measures of sleep problems (insomnia and sleep quality); a global measure of burn-out, along with two of its subindices, fatigue and cognitive weariness; a measure of work-induced fatigue during free time; and a measure of somatic symptoms. It was hypothesised that there would be changes in the primary measures reflecting improvements in well-being. In addition, a set of secondary outcomes were examined, focusing on factors that could help account for changes in the primary outcomes, namely: perceived stress; two remaining subscales of burn-out (listlessness, tense) and dysfunctional attitudes about sleep.

## Methods

### Design

A parallel randomised control trial was designed to include 100 participants in each group (intervention and wait list control) to detect moderate effect sizes (Cohen’s d=0.5) resulting in a power of 0.94. Excel generator for random allocation to groups was used by the research team. Based on a previous feasibility study, adjustments to the process of random group allocation were made if many nurses from the same ward were initially allocated to one group.^20^ Adjustments were also made for participants who were randomised to the intervention group but knew that they could not attend the group sessions. They were moved to the control group and replaced by a random participant from the control group. Adjustments were made for 24 participants. Masking was not applicable. After the follow-up measure the control group received the intervention.

### Participants and data collection

RNs with less than 12 months’ work experience were eligible to participate. Participants were recruited at eight Swedish hospitals within the induction programmes for newly graduated RNs at seven of the hospitals. One hospital did not have such a programme and so the RNs there were recruited via managers. The intervention was tested in ten subgroups with 5–13 participants in each, between 2017 and 2018. All participants signed an informed consent before entering the study and were thereafter enrolled in the study by the research team.

Digital questionnaires assessing the outcomes were sent to participants by email about 1 month before entering the intervention (baseline), 1 month after the intervention (postintervention) and at 6 months after the intervention (follow-up). Participants who had attended any of the group sessions received a short questionnaire, approximately 2 weeks after each session, evaluating the use of recovery strategies from the programme.^23^ As from the fourth subgroup, a global evaluation questionnaire was distributed after the intervention (in total 62 participants).

### Intervention

The intervention was a group-administered proactive recovery programme focusing on enhancing beneficial strategies for sleep and recovery as a means of mitigating the impact of work stress and shift work.^23^ The programme was developed by MS (certified psychologist, PhD) and AD (PhD) and included three group sessions (2,5 hours), with one session every second week (ie, 4 weeks from the first session to the third), during work hours at the hospitals. MS trained AD and ME (Bachelor of applied psychology) in delivering the recovery programme. Seven subgroups were led by MS together with AD and/or ME, three subgroups were led by AD and ME.

The intervention was based on CBT and motivational interviewing techniques.^13 16 20 24^ The ‘sleep formula’—that is, the influence of stress, homoeostatic and circadian factors on sleep—was used as a pedagogical approach to summarise research-based knowledge about what regulates sleep. The sessions had three main focuses: (1) unwinding from stress, including detachment from thoughts of work during free time; (2) supporting sleep in relation to homoeostatic and circadian processes; and (3) handling fatigue and increasing recovery behaviours (see table 1). Psychoeducative elements were interspersed with group discussions and exercises. Participants were encouraged to reflect on their habitual behaviours connected to sleep and recovery and possible alternatives. Between sessions, the participants were encouraged to try strategies or behaviour changes of their choice, with the aim of enhancing sleep and recovery. During the second and third sessions, participants reflected on the experience of trying new strategies. All participants received written material covering the content of each session, as well as online access to an adapted version of a biomathematical model (ArturNurse). ArturNurse evaluated fatigue risk levels based on their work schedules^25^ and provided suggestions of strategies from the programme on how to optimise sleep in relation to different shifts. See online supplemental file 1 for more detail about the intervention.

10.1136/oemed-2021-107789.supp1 Supplementary data

**Table 1:** Content of group sessions (I–III)

Session	Content	Strategies participants were encouraged to try
I. Unwinding from stress	The sleep formula Stress factors and stress reactions at work CBT-model: Analysis of behaviour in stressful work situations Unwinding routines before bedtime Mindfulness, focus on the present moment Body scan exercise	Observe behaviours in stressful work situation and reflect on alternatives Practice focusing on the present moment Unwinding bedtime routine Body scan
II. Promoting sleep according to homoeostatic and circadian factors	Follow-up from session I Routines for leaving work Homoeostatic processes regulating sleep Circadian processes regulating sleep How work hours interact with sleep regulating factors	Routine for leaving work Personal goal for supporting sleep related to the homoeostatic and circadian processes Evaluating work hours using the ArturNurse webtool
III. Handling fatigue by increasing recovery behaviours	Follow-up from session II Cognitive, physical and emotional fatigue Balance between activity and rest Short relaxation exercise Recovery behaviour on and off work Activities boosting energy	Practice recovery behaviours at work Engaging in activities boosting energy during free time Practice short relaxation

CBT, cognitive–behavioural therapy.

### Background measures

In the baseline questionnaire, participants reported gender (male, female, other), age (years), duration of working as a nurse (months), type of shift schedules, if they took any medication (yes/no), and frequency of the use of sleep medication, central stimulants, sedatives, opioid analgesics or other pain killers (1 never, 5 every day).

## Primary outcomes

### Sleep

Insomnia symptoms during the last month were measured with the Insomnia Severity Index (ISI; 0 no problems—4 severe problems).^26^ A sum score was calculated (Cronbach’s alpha=0.84), 15 or higher indicates clinical insomnia. A sleep quality index was calculated based on the mean of four items from the Karolinska Sleep Questionnaire (KSQ)^27^ (Cronbach’s alpha=0.77) rating the frequency of sleep problems (1 always—6 never).

### Burn-out, fatigue and cognitive weariness

Burn-out symptoms during the last month were measured with the Shirom-Melamed Burn-out Questionnaire (SMBQ) consisting of 22 items (1 almost never—7 almost always).^28 29^ A global mean score was calculated (Cronbach’s a=0.95), and the two indices: ‘fatigue’ (Cronbach’s a=0.89) and ‘cognitive weariness’ (Cronbach’s a=0.94).

### Work-Induced fatigue

Work-induced fatigue during free time was measured with the Work Interference with Personal Life index (WIPL) from the Work Home Interference scale^30^ based on the mean of four items (Cronbach’s alpha=0.90) measuring the extent to which work related fatigue affects free-time (1 not at all—5 almost all the time). Scores of ≥3.5 indicates work-home interference.^31^

### Somatic symptoms

Somatic symptoms were measured with the Somatic Symptom Scale-8 (SSS8), which assesses the experience of eight somatic symptoms (eg, headache, stomach problems, back pain) during the last 7 days (0 not at all—4 much). A sum score was calculated (Cronbach’s alpha=0.75). Scores 8–11 indicate a medium somatic symptom burden, 12–15 indicate high and 16–32 indicate very high.^32^

## Secondary outcomes

### Perceived stress

Perceived stress during the last month was measured with the Perceived Stress Scale (PSS) consisting of 10 items (0 never—4 very often). A global mean score was calculated (Cronbach’s alpha=0.88).^33^

### Tension and listlessness

The indices ‘listlessness’ (Cronbach’s a=0.82) and ‘tense’ (Cronbach’s a=0.73) from the SMBQ were calculated.^28^

### Dysfunctional beliefs and attitudes about sleep

Dysfunctional beliefs and attitudes about sleep were measured through the Dysfunctional Beliefs and Attitudes about Sleep scale (DBAS-10).^34^ In the original version, the degree of agreement with 10 statements is measured on a Visual Analogue Scale between 0 and 100. However, due to technical problems, data from the first three subgroups (in total 46 participants) were excluded from the analyses, while as from the fourth sub-group a ten point scale (0 do not agree—10 do fully agree; Cronbach’s alpha=0.80) was used.

## Statistical methods

Longitudinal analysis of mean response profiles,^35^ with time coded as a categorical variable (in order to account for possible non-linear relationships), was performed using the mixed model procedure in IBM SPSS Statistics V.26. Maximum likelihood was used to estimate the parameters (using all available data) under the assumption that incomplete data were missing at random. A significant group-by-time interaction was interpreted as reflecting differential patterns of change between the groups over time. Calculations of effect sizes based on group mean differences postintervention and at follow-up were calculated on model-based estimated means and SD where a Cohen’s d around 0.5 was considered as moderate and around 0.2 as small.

## Results

**Figure 1:**
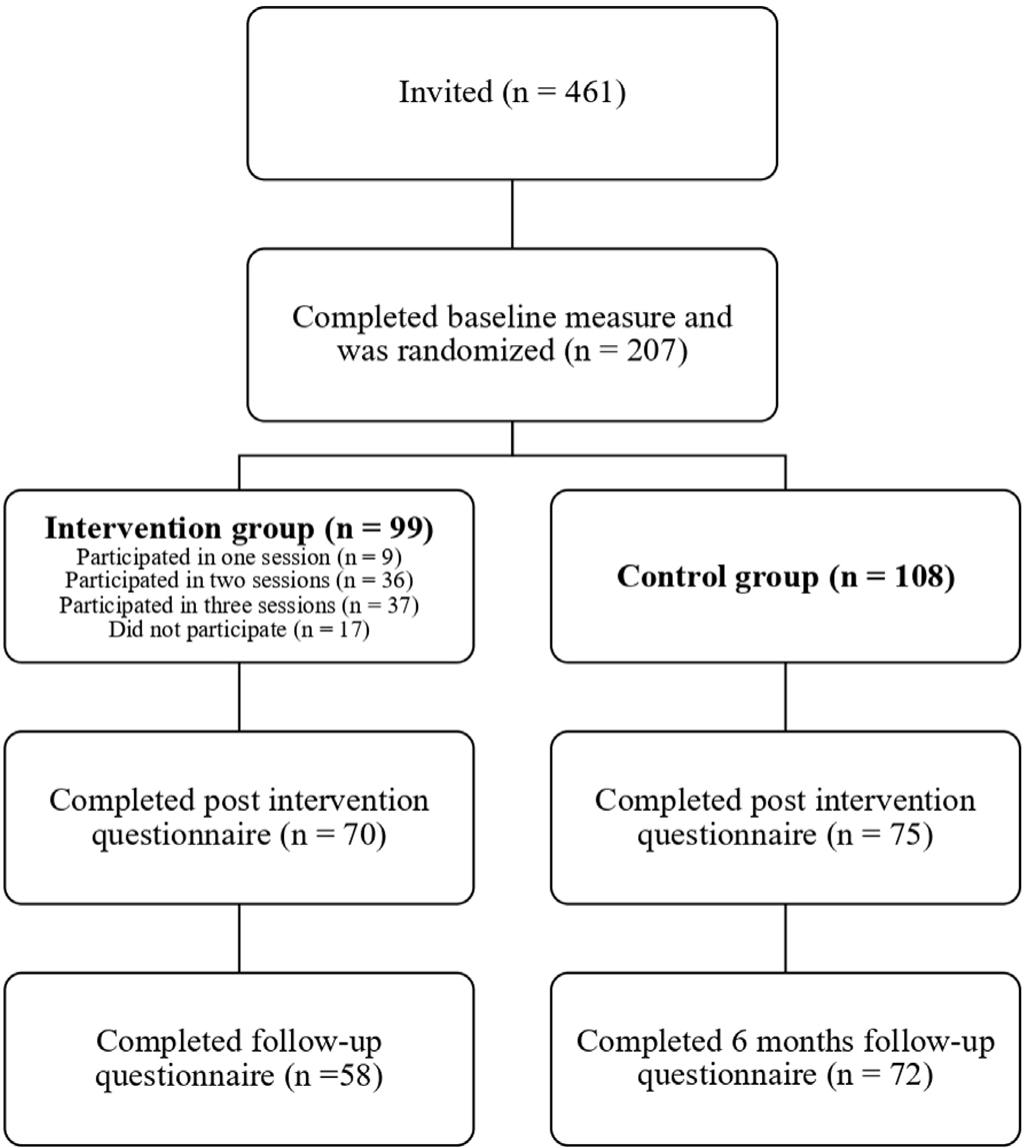
Participant flow chart.

Figure 1 shows the participant flow chart, showing how the final sample was arrived at. Of 461 invited new RNs, 207 (45%) signed up for the study.

### Baseline data

The intervention and control group consisted of 84.7% and 90.7% women, respectively. The average age in the intervention group was 27.5±5.3 and 27.0±5.1 in the control group. The average time of employment was 2.8±2.1 months in the intervention group and 3.3±2.7 months in the control group. Most participants (73%) had a rotating morning and evening shift schedule, and almost one fifth (19%) had a rotating morning, evening and night shift schedule. No significant differences were observed between the two groups at baseline for any of the background variables or any of the outcome measures at baseline (see online supplemental file 2).

10.1136/oemed-2021-107789.supp2 Supplementary data

### Sleep, burn-out, fatigue and somatic symptoms

**Figure 2:**
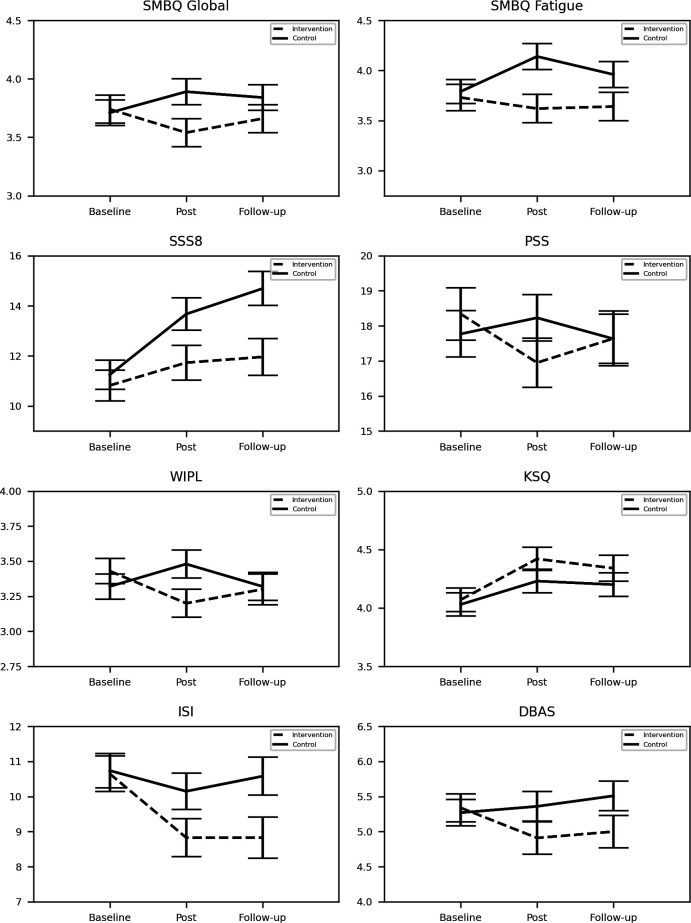
Mean values and SEs in intervention and control group at baseline, post and follow-up measures. DBAS, Dysfunctional Beliefs and Attitudes about Sleep, 0 do not agree–10 do fully agree; ISI, Insomnia Severity Index, 0–28 severe problems; KSQ, Karolinska Sleep Questionnaire, 1 always–6 never; PSS, Perceived Stress Scale, 0 never–40 very often; SSS8, Somatic Symptom Scale-8, 0–32 very high somatic symptom burden; SMBQ, Shirom-Melamed Burn-out Questionnaire, 1 almost never–7 almost always; WIPL, Work Interference with Personal Life, 1 not at all–5 almost all the time.

Results relating to the primary outcomes are shown in table 2 and figure 2. Insomnia symptoms (ISI) and sleep quality (KSQ) showed no significant group by time interaction.

**Table 2:** Estimated group means and tests of effects, taken from a multilevel analysis of the primary outcome measures using an intention to treat approach

	Estimated group means						Tests of effects								Cohen’s d between groups	
	Intervention			Control				Group		Time			Group * Time			
	Mean	SE	95% CI	Mean	SE	95% CI	F	P value	df	F	P value	df	F	P value	Post	Follow-up
**ISI**							2.88	0.09	185.39	6.73	0.00	148.41	2.57	0.08	0.30	0.39
Baseline	10.65	0.51	9.64 to 11.66	10.74	0.49	9.78 to 11.71										
Post	8.83	0.54	7.76 to 9.90	10.15	0.52	9.12 to 11.19										
Follow-up	8.83	0.59	7.66 to 10.00	10.58	0.54	9.51 to 11.65										
**KSQ**							1.02	0.31	196.14	11.25	0.00	153.04	0.75	0.47	−0.22	−0.17
Baseline	4.07	0.10	3.87 to 4.27	4.03	0.10	3.84 to 4.22										
Post	4.42	0.10	4.22 to 4.62	4.23	0.10	4.04 to 4.43										
Follow-up	4.34	0.11	4.11 to 4.56	4.20	0.10	4.00 to 4.40										
**SMBQ Global**							1.45	0.23	190.01	0.17	0.84	149.74	4.03	0.02	0.36	0.19
Baseline	3.74	0.12	3.51 to 3.97	3.71	0.11	3.49 to 3.93										
Post	3.54	0.12	3.30 to 3.77	3.89	0.11	3.67 to 4.12										
Follow-up	3.66	0.12	3.43 to 3.90	3.84	0.11	3.63 to 4.06										
**SMBQ Fatigue**							3.90	0.05	187.18	1.05	0.35	147.17	3.87	0.02	0.45	0.30
Baseline	3.73	0.13	3.48 to 3.98	3.79	0.12	3.55 to 4.02										
Post	3.62	0.14	3.35 to 3.88	4.14	0.13	3.89 to 4.40										
Follow-up	3.64	0.14	3.37 to 3.91	3.96	0.13	3.71 to 4.21										
**SMBQ Cognitive**							0.10	0.76	189.40	0.43	0.65	147.13	3.53	0.03	0.24	0.03
Baseline	3.47	0.15	3.17 to 3.77	3.33	0.14	3.04 to 3.61										
Post	3.16	0.16	2.85 to 3.47	3.47	0.15	3.18 to 3.77										
Follow-up	3.37	0.16	3.06 to 3.69	3.37	0.15	3.08 to 3.66										
**WIPL Fatigue**							0.31	0.58	184.30	0.44	0.65	149.17	5.37	0.01	0.33	0.02
Baseline	3.43	0.09	3.25 to 3.62	3.32	0.09	3.14 to 3.49										
Post	3.20	0.10	2.99 to 3.40	3.48	0.10	3.29 to 3.68										
Follow-up	3.30	0.11	3.08 to 3.51	3.32	0.10	3.13 to 3.52										
**SSS8**							4.48	0.04	176.33	16.30	0.00	132.72	3.81	0.03	0.37	0.49
Baseline	10.82	0.61	9.61 to 12.03	11.25	0.58	10.11 to 12.39										
Post	11.73	0.69	10.36 to 13.09	13.67	0.65	12.38 to 14.95										
Follow-up	11.96	0.73	10.51 to 13.41	14.69	0.68	13.36 to 16.02										

Factor labels in tests of effects: Group=Intervention vs Control, Time=Baseline vs Post vs Follow-up, Group*time=interaction term. Df (df) for Time and Group*time are identical.

ISI, Insomnia Severity Index, 0–28 severe problems; KSQ, Karolinska Sleep Questionnaire, 1 always— 6 never; Mean, modelled mean values; SMBQ, Shirom-Melamed Burn-out Questionnaire, 1 almost never—7 almost always; SSS8, Somatic Symptom Scale-8, 0–32 very high somatic symptom burden; WIPL, Work Interference with Personal Life, 1 not at all—5 almost all the time.

Symptoms of burn-out (SMBQ) showed significant group by time interactions for both the global score and for the indices ‘fatigue’ and ‘cognitive weariness’. Post hoc analysis showed the same general pattern for all three outcomes; the intervention group reported less symptoms postintervention (small to moderate effect sizes), but did not differ from the control group at follow-up.

Ratings of work-induced fatigue (WIPL) during free time showed a significant group by time interaction, where the intervention group reported less fatigue postintervention (small effect size), but not at follow-up.

Ratings of somatic symptoms were relatively stable over time in the intervention group, whereas the control group reported increased somatic symptoms (SSS8). This was reflected in the significant group by time interaction and in significant differences in the post hoc tests postintervention and at follow-up, with higher somatic symptoms observed in the control group (small to moderate effect sizes).

### Perceived stress, tension, listlessness, DBAS

Results relating to the secondary outcomes are shown in table 3 and figure 2. No significant group by time interactions were found for the ratings of perceived stress (PSS), for either of the SMBQ indices ‘tense’ or ‘listlessness’, or for beliefs and attitudes about sleep (DBAS).

**Table 3:** Estimated group means and tests of effects, taken from a multilevel analysis of the secondary outcome measures using an intention to treat approach

	Estimated group means						Tests of effects								Cohen’s d between groups	
	Intervention			Control			Group			Time			Group * Time			
	Mean	SE	95% CI	Mean	SE	95% CI	F	P value	df	F	P value	df	F	P value	Post	Follow-up
**PSS**							0.08	0.78	175.63	0.52	0.60	144.83	2.24	0.11	0.22	0.01
Baseline	18.34	0.75	16.87 to 19.81	17.77	0.66	16.48 to 19.07										
Post	16.95	0.70	15.56 to 18.34	18.23	0.66	16.93 to 19.53										
Follow-up	17.64	0.78	16.10 to 19.18	17.63	0.70	16.25 to 19.01										
**SMBQ Tense**							0.05	0.82	191.85	2.78	0.07	148.94	0.89	0.41	0.03	0.14
Baseline	3.83	0.13	3.58 to 4.08	3.75	0.12	3.51 to 3.99										
Post	3.66	0.14	3.38 to 3.94	3.69	0.14	3.42 to 3.96										
Follow-up	3.82	0.15	3.54 to 4.11	3.98	0.13	3.72 to 4.24										
**SMBQ Listlessness**							1.99	0.16	190.88	0.59	0.56	150.50	2.89	0.06	0.39	0.15
Baseline	4.06	0.13	3.81 to 4.31	4.11	0.12	3.88 to 4.35										
Post	3.81	0.12	3.57 to 4.05	4.21	0.12	3.98 to 4.45										
Follow-up	3.99	0.14	3.72 to 4.27	4.16	0.13	3.91 to 4.41										
**DBAS**							1.32	0.25	153.20	1.15	0.32	129.95	2.94	0.06	0.26	0.32
Baseline	5.34	0.20	4.94 to 5.74	5.27	0.19	4.89 to 5.65										
Post	4.91	0.23	4.46 to 5.36	5.36	0.21	4.94 to 5.79										
Follow-up	5.00	0.23	4.54 to 5.45	5.51	0.21	5.10 to 5.93										

Factor labels in tests of effects: Group=Intervention vs Control, Time=Baseline vs Post vs Follow-up. Df (df) for Time and Group*time are identical.

DBAS, Dysfunctional Beliefs and Attitudes about Sleep, 0 do not agree—10 do fully agree; Mean, modelled mean values; PSS, Perceived Stress Scale, 0 never—40 very often; SMBQ, Shirom-Melamed Burn-out Questionnaire.

## Compliance and programme evaluation

Unwinding bedtime routines were used by 95% of those who attended any of the group sessions (N=82), routines for leaving work by 87%, relaxation exercise by 86%, activities promoting recuperation by 75% and body scan meditation by 74% (response rates 91%–94%). Recovery behaviours during work and free time were used by 82% and 80%, respectively, and the short relaxation exercise by 70%, whereas the webtool ArturNurse was used by 21% (response rates 60%–78%). Strategies related to homoeostatic or circadian processes were used by 78% (response rate 45%). All respondents (100%) reported that they would recommend the programme to others, and 98% rated the programme as good or very good. The majority, 90%, reported that they would use the strategies in the future, and 8% that they might do so (79% response rate).

## Discussion

This study examined whether a proactive intervention for newly graduated RNs, supporting strategies for the enhancement of sleep and recovery in relation to work stress and shift work, could prevent negative development of sleep problems, burn-out, fatigue and somatic symptoms. The results indicated a preventive effect on somatic symptoms, as the intervention group showed stable ratings for these symptoms, while the control group showed increased somatic symptoms over time. Further, promising effects were seen on burn-out measures and on work-induced fatigue during free time at postintervention. However, these latter effects did not persist at follow-up 6 months later.

The intervention group showed lower global burn-out scores compared with the control group, as well as lower scores on the indices ‘fatigue’ and ‘cognitive weariness’ postintervention. However, the effects on these burn-out measures did not persist at follow-up 6 months later. It remains to be determined whether a booster session or if changes to the programme could contribute to a longer-lasting effect.

Another promising finding was that the intervention group reported less work-induced fatigue during free time, postintervention. Previous research indicates that nurses’ work-home balance suffers at the start of their career.^36^ It is possible that the intervention helped the nurses to detach from work stress during free time, thereby enabling them to achieve a better quality of recovery. An improved work-home balance suggests that the recovery programme may strengthen important preconditions for a sustainable working life and counteract the so-called ’recovery paradox’.^10^

There was no significant interaction effect in the analysis of sleep quality or insomnia symptoms. However, the results showed trends towards interaction, suggesting less insomnia symptoms and decreased DBAS in the intervention group, thus following the same pattern as for the other outcome variables. A possible explanation for the lack of significant effects on sleep quality might be that work requirements (eg, timing of shifts) constrain the extent to which sleep can be altered. More fine-grained analysis, such as day-to-day comparisons, might capture a more nuanced picture of sleep quality or other sleep parameters. It is also important to note that the recovery programme was not consistent with a regular CBT-protocol for insomnia. Rather, a preventive approach was taken with participants being included regardless of whether insomnia was present. This study is therefore not comparable to other therapeutic intervention studies^14 15 37^ in which participants were included on the basis of sleep problems. The proactive approach of the intervention may also partly explain why the effect sizes were only small to moderate.

Previous studies have reported impaired self-rated health among new RNs during the transition from education into working life.^38^ Therefore, the present finding that somatic symptoms did not increase over time for nurses in the intervention group, but did so for the control group, is important, indicating a preventive effect of the recovery programme on somatic symptoms.

While the intervention was effective in reducing fatigue and preventing somatic symptoms, it had no significant effect on the secondary outcomes of perceived stress (PSS), listlessness or tension (SMBQ indices). This may imply that both groups reacted similarly to the challenges they face as new nurses. Notably, the intent of the recovery programme was not to decrease stress reactions per se, but to improve the quality of recovery and increase the use of recovery behaviours—in line with the theoretical perspective that stress is not necessarily harmful as long as there is sufficient recovery.^4^

The broad approach of the recovery programme, targeting factors regulating sleep and recovery (unwinding from stress, supporting sleep according to homoeostatic and circadian factors, increasing recovery behaviours), may have helped counteract fatigue development. Fatigue is a signal of the need for recovery and so fatigue should decrease when recuperation is strengthened.^2 39^ Our results point to the value of a holistic approach to recovery.

Major strengths of the intervention were that it was short, proactive and proved feasible in a working life context. Despite that only 37% attended all three sessions, the programme achieved high measured compliance. Compliance may have been boosted by the participants receiving written materials after each session.

The programme included a wide range of strategies aimed at enhancing both sleep and other forms of recovery, possibly making it easier for participants to find strategies to apply. On the other hand, the intervention’s broad approach limits the possibility to explain specific mechanisms behind the results.

Some limitations are worth noting. The sampling may have been biased by self-selection into the study, towards nurses with a high motivation to participate. Mandatory participation might have produced different results. Moreover, we cannot draw any conclusions as to whether the recovery programme would be feasible or effective in other occupational groups, or for participants with more extreme workloads, or with clinically significant sleep or burn-out problems. Notably, only 37% attended all three group sessions, highlighting the need to develop approaches to increase attendance at group sessions. Another limitation was the variation in response rates regarding the use of strategies within the programme. A deeper understanding of how the different strategies have been used will be examined in analysis of follow-up interviews with participants and reported in future publications.

The results merit further evaluation of the recovery programme as a part of induction programmes for new RNs. Future studies should examine the feasibility of implementing the recovery programme in nursing education, or whether it could be adapted for nurses who are further into their career or for other professional groups. Enabling shift workers to cope with their demanding work hours makes strong economic sense, as it may help reduce turnover and absenteeism rates, to the mutual benefit of employees and employers.^40^ Nevertheless, organisations still have a responsibility to provide healthy work environments and work schedules that enable sufficient recuperation on and between shifts, in order to promote sustainable work conditions.

To conclude, a short, proactive, group-administered recovery programme was helpful in strengthening recovery for newly graduated RNs, by way of preventing somatic symptoms, and reducing burn-out symptoms and work-induced fatigue, suggesting recovery as a key factor in the prevention of negative health consequences of work stress.

## Data Availability

No data are available. Data obtained for the study will not be accessible to others.
