# Supplementary material for: Randomised control trial of a proactive intervention supporting recovery in relation to stress and irregular work hours: effects on sleep, burn-out, fatigue and somatic symptoms
Source: Occup Environ Med. 2022 Jan 24;79(7):460–8. doi: 10.1136/oemed-2021-107789 (PMC9209685; doi:10.1136/oemed-2021-107789)
Supplement: Supplementary data [file oemed-2021-107789supp001.pdf]

Supplement 1 - The intervention

The intervention was a proactive group-administered recovery programme based on research on stress, sleep and work hours, and cognitive behavioral therapy (CBT) (e.g. reference 13, 16, 20, 24 in the manuscript). CBT methods used were for example behavior analysis, mindfulness, acceptance and exposure strategies, and behavioural change in valued directions. The general coaching approach from the group leaders was influenced by motivational interviewing (MI), including open-ended questions and empathic listening aimed at evoking the participants' own willingness to try new behaviors or strategies of their choice.

The programme included three group sessions (2.5 hours each) with a two-week interval. During the sessions, psychoeducative elements were interspersed with group discussions and exercises. Focus was on possible helpful strategies for improving recovery when working as a newly registered nurse. Between sessions, participants were encouraged (i.e. it was voluntary) to try different behavioral changes and strategies, based on what was discussed during the sessions, to promote recovery. Participants were encouraged to share their experiences the following session, and reflect on how to continue. The third session could not be followed up, but participants were encouraged to continue trying the different strategies presented in all three sessions.

The 'sleep formula'– i. e. the influence of stress, homeostatic and circadian factors on sleep – was used as a pedagogical approach to summarize research-based knowledge about what regulates and disturbs sleep. Based on that knowledge, a mixture of different strategies was presented on how to promote sleep and recovery in relation to work-related stress and irregular working hours. The intervention included three themes, one in focus each session: 1) unwinding from stress, 2) promoting sleep according to homeostatic and circadian factors, and 3) handling fatigue by increasing recovery behaviors.

The strategies presented focused on how to establish unwinding routines before bedtime as well as helpful routines for leaving work, in order to let go of stressful thoughts of work during free time. Mindfulness strategies were presented as a tool for focusing on the present moment at work, as well as for unwinding before bedtime. Strategies for promoting sleep were presented and discussed as establishing routines in line with the homeostatic and circadian processes, e. g. changing bedtimes in order to build up enough sleep pressure, or to time unwinding routines and bedtimes with the circadian rhythm, or to expose oneself to daylight at optimal times of day, in order to strengthen the circadian regulation of sleep. As the participants worked shifts they were encouraged to use an 'anchor sleep' in accordance with the circadian rhythm. (i. e. a sleep schedule where a core part of the sleep overlap, workdays and rest days). Different strategies for handling fatigue at work were presented and discussed, for example taking breaks, focusing on the present moment, or using alternative behaviors in stressful situations. The participants were encouraged to try to use daily recovery behaviors of their own choice, e. g. physical activity, listening to music, meet with friends, or just take a deep breath, on and off work.

In addition, the participants had access to a webtool, ArturNurse (available at http://nurse.arturcloud.com), supporting the evaluation of work schedules from a recovery perspective. It was based on the three-process model (reference 25 in the manuscript) and the interface was developed to fit the purpose of the study. ArturNurse was based on estimated sleep length between shifts and identified shifts with a higher risk for fatigue. The shifts were color coded as green (low risk for fatigue), yellow (somewhat elevated risk for fatigue), orange (risk for fatigue) and red (high risk for fatigue). When placing the marker over a yellow, orange or red shift a short list referring to the strategies from the programme appeared (e.g. unwind before bed time, get daylight in the morning, take a nap before night shifts, etc).
