# Supplementary material for: Randomised control trial of a proactive intervention supporting recovery in relation to stress and irregular work hours: effects on sleep, burn-out, fatigue and somatic symptoms
Source: Occup Environ Med. 2022 Jan 24;79(7):460–8. doi: 10.1136/oemed-2021-107789 (PMC9209685; doi:10.1136/oemed-2021-107789)
Supplement: Supplementary data [file oemed-2021-107789supp002.pdf]

Supplementary table 2. Independent t-test and chi- square test of differences between intervention and control groups at baseline

	t/χ^2	df	P
**Background information**			
Age	t= 0.745	198	0.457
Gender	χ^2= 0.18	1	0.18
Months of employment	t= -1.351	205	0.178
Medication (yes/no)*	χ^2= 0.889	1	0.502
Sleep medications#	t= -0.668	81	0.506
Sedatives#	t= -1.205	80	0.232
Opioid analgesics#	t= 0.102	80	0.919
Other painkillers#	t= -0.066	83	0.947
**Primary outcomes**			
ISI^	t= -0.095	201	0.924
KSQ	t= 0.293	204	0.77
SMBM Global	t= 0.157	199	0.875
SMBM Fatigue	t= -0.323	199	0.747
SMBM Cognitive	t= 0.699	199	0.485
WIPL	t= 0.89	201	0.375
SSS8	t= -0.364	154	0.716
**Secondary outcomes**			
PSS10	t= 0.702	189	0.483
SMBM Listlessness	t= -0.324	199	0.746
SMBM Tense	t= 0.464	199	0.643
DBAS	t= 1.056	203	0.292

*45 % used medication.

1 never, 5 every day. The prevalence of using medication every week (4) or every day (5) were for sleep medication 6 %; central stimulants 0 %; sedatives 2 %; opioid angelica 2 %; other pain killers 26 %.

^About one fifth (22 %) of the participants had clinical insomnia problems.

ISI- Insomnia Severity Index, 0-28 severe problems; KSQ - Karolinska Sleep Questionnaire, 1 always - 6 never; SMBQ - Shirom-Melamed Burnout, 1 almost never - 7 almost always; WIPL - Work Interference with Personal Life, 1 not at all - 5 almost all the time; SSS8 - Somatic Symptom Scale-8, 0-32 very high somatic symptom burden; PSS – Perceived Stress Scale, (0 never - 4 very often); DBAS - Dysfunctional Beliefs and Attitudes about Sleep, 0 do not agree - 10 do fully agree.
